# Vermicomposting manure-paper mixture with igneous rock phosphate enhances biodegradation, phosphorus bioavailability and reduces heavy metal concentrations

**DOI:** 10.1016/j.heliyon.2018.e00749

**Published:** 2018-08-22

**Authors:** Lushian Tapiwa Mupondi, Pearson Nyari Stephano Mnkeni, Pardon Muchaonyerwa, Hupenyu Allan Mupambwa

**Affiliations:** a Department of Agronomy, Faculty of Science and Agriculture, University of Fort Hare, P. Bag X1314, Alice, South Africa; b School of Agricultural, Earth and Environmental Sciences, University of KwaZulu-Natal, P. Bag X01, Scottsville, South Africa; c Sam Nujoma Marine and Coastal Resources Research Centre, Sam Nujoma Campus, University of Namibia, P. O. Box 462, Henties Bay, Namibia

**Keywords:** Agriculture

## Abstract

In organic soil fertility management, rock phosphate (RP) is gaining momentum as an acceptable phosphorus source, though much of this P is not bioavailable for plant uptake, particularly in igneous RP. This study evaluated the nutrient solubilization, biodegradation and heavy metal concentration when cow dung – waste paper mixture amended with increasing rates of igneous RP was vermicomposted with *E. fetida*. The cow dung was optimized to a C/N ratio of 30 using waste paper and amended with RP to provide 0%; 2%; 4% and 8% of elemental phosphorus on a dry w/w basis. Incorporation of RP at 2% and 8% P enhanced compost biodegradation resulting in a 12% and 22% significantly (*P* < 0.001) lower final C/N ratio, respectively, compared to the control; together with higher humification parameters. Amending the cow dung – waste paper mixture with 2%, 4% and 8% P as rock phosphate, resulted in a 39%; 50% and 65% more resin extractable P, respectively, relative to the control. Similarly, the bicarbonate extractable P, which represents the bioavailable P fraction, increased consistently by 19%; 28% and 33% following 2%, 4% and 8% RP application, respectively. Though incorporation of RP initially resulted in increased heavy metal levels, reductions of 40%; 35%; 35%; 40% and 45% for Cr, Cu, Cd, Pb and Zn, respectively, were observed in the 8% RP treatment after 8 weeks, due to the presence of earthworms. Vermicomposting with *E. fetida* significantly reduced heavy metals to levels below the maximum permissible concentration of potentially toxic elements in soils after 8 weeks. This study demonstrates the potential of optimized vermicomposting with igneous RP for generating nutrient rich organic fertilizers.

## 1 Introduction

Phosphorus (P) is an essential plant nutrient, affecting major biochemical processes within plants, and therefore affects crop production (Aria et al., 2010; Sharma et al., 2013). Though P is found abundant in some soils, it is mostly precipitated or insoluble through its association with other elements and thus not readily available for plant uptake (Odongo et al., 2007). Due to its low levels in most soils, available P needs to be supplemented through use of inorganic chemical fertilizers, which represents a major cost in crop production (Alamgir et al., 2012). The economic and environmental challenges associated with the use of chemical fertilizers, such as eutrophication and diminishing P reserves, have driven researchers to search for alternative P sources. Several researchers have evaluated the potential of P rich industrial waste materials such as fly ash in soil improvement, though fly ash has not yet been accepted in organic soil fertility management as a nutrient source (Mupambwa and Mnkeni, 2016, 2018). Conversely, direct application of rock phosphate (RP) has been promoted as one such alternative but its effectiveness is highly dependent on the solubility of the RP in soil (Mupambwa and Mnkeni, 2018). In addition, most research on RP has only focused on using readily soluble sedimentary RP with limited research on the highly insoluble igneous RP.

Rock phosphates predominating in southern Africa are of igneous origin, with the P bearing apatite minerals having limited isomorphous substitution, which limits their reactivity and solubility (MIF, 2003). The low bioavailability of the total P in igneous RP, is a major limitation to the use of RP as a P source and approaches such as phospho-composting could increase the bioavailability of P which could be of great value in organic agriculture (Alamgir et al., 2012; Edwards et al., 2010; Mupambwa and Mnkeni, 2018). Thermophilic composting and vermicomposting are processes that can be used to enhance the plant available nutrients content and thus acceptability of RP as an alternative P source in agriculture. Vermicomposting has gained momentum as an environmentally friendly method of converting organic waste materials into valuable fertilizers, with the capacity to also solubilize nutrients within inorganic materials like rock phosphate (Edwards et al., 2010; Adhami et al., 2014).

Organic acids released during vermicomposting result in the solubilization of P from P-bearing minerals, thus enhancing P bio-availability. Unlike chemical fertilizers, vermicomposts have several benefits such as improving both the bio-chemical and physical properties of the soil whilst increasing health related secondary metabolites in plants (Goswami et al., 2017; Das et al., 2018). Although several researchers have looked at the potential of composts and vermicompost on P release from rock phosphate, much of the work was based on amendment of mature composts or non-optimized vermicomposts, reducing the potential mineralization benefits of the composting processes (Kumar and Singh, 2001; Aria et al., 2010; Busato et al., 2012). Research by Ndegwa and Thompson (2000) and Mupondi (2010) indicated the optimum C/N ratio for effective vermicomposting as 30. Incorporating waste paper into cow dung has been suggested as one way of optimizing the C/N ratio of organic materials. The use of waste paper as a carbon base has been indicated as a critical recycling opportunity especially in developing countries where only about 19% of paper is recycled with the rest of it being burnt (Gupta and Garg, 2009; Mupambwa and Mnkeni, 2015). However, there are very few studies that have looked at the nutrient mineralization of rock phosphate, incorporated at different levels, under optimized vermicomposting conditions (Mupambwa and Mnkeni, 2018). While the resultant phospho-composts could be of great agricultural value, their effectiveness could be affected by the concentration of heavy metals from some rock phosphates.

Rock phosphate contains variable quantities of heavy metals like cadmium (Cd), chromium (Cr), mercury (Hg) and lead (Pb) and radioactive elements, like uranium (U) that are toxic to human and animal health (Mortvedt and Sikora, 1992). This is especially true for Cd, which is usually bound with P in the apatite structure (Sery and Greaves, 1996) and is thus concomitantly released with P during solubilization. Earthworms have, however, been reported to accumulate heavy metals in their bodies and their involvement in vermicomposting of wastes could reduce the amounts of heavy metals present in the final vermicomposts (Dai et al., 2004; Goswami et al., 2016). A protein called metallothionein, which plays a critical role in regulation of heavy metal ion chemistry within cells, has been reported to be produced within the gut of earthworms upon exposure to heavy metals (Goswami et al., 2016; Usmani et al., 2017). However, there are no studies that have focused on changes in heavy metals during vermicomposting of rock phosphate, and on influence of the presence of earthworms on heavy metal concentrations. It is essential to understand more the biodegradation, nutrient release and changes in heavy metal concentrations during vermicomposting of cow dung – waste paper mixture amended with rock phosphate (Usmani et al., 2017). Thus, the objectives of this study were to evaluate the influence of different inclusion levels of igneous rock phosphate during vermicomposting of cow dung-waste paper mixture on (i) phosphorus and nitrogen bioavailability, compost maturity, and microbial biomass carbon; and (ii) heavy metal concentration in compost and bioaccumulation in earthworms.

## 2 Materials and methods

### 2.1 Materials for vermicomposting

The study was carried out at the vermicomposting research unit at the University of Fort Hare, Alice Campus (32°46′ S and 26°50′ E) located in the Eastern Cape Province of South Africa. Cow dung used in the study was obtained from the Keiskammahoek Dairy Project located about 60 km North East of Alice. Following collection, the cow dung was crushed to remove large lumps, air dried, and stored under shed in a dry area. Shredded waste paper was obtained from the Duplicating Center of the University of Fort Hare, Alice Campus, while other waste paper was collected from offices at the University, and shredded. Representative samples of the cow dung and waste paper were analyzed for selected chemical properties. For pH and electrical conductivity, the shredded paper and ground cow dung were separately determined in a 1:10 suspension (w v^−1^) using pH and conductivity meters as described by Unuofin and Mnkeni (2014). The selected chemical properties of the cow dung and waste paper are shown in Table 1.

**Table 1:** Selected chemical parameters of the materials used in the vermicomposting experiment.

Parameter	Cow dung	Waste paper
pH	7.8 ± 0.1	8.2 ± 0.3
Electrical conductivity (mS m^−1^)	440 ± 2.0	0.18 ± 0.1
Total C (g kg^−1^)	321 ± 3.0	370 ± 5.3
Total N (g kg^−1^)	24 ± 2.5	3 ± 0.6
C:N ratio	13 ± 0.6	123 ± 3.7
Total P (g kg^−1^)	2.9 ± 0.4	0.5 ± 0.1
C:P	110 ± 50	740 ± 15.5
Ash (g kg^−1^)	379 ± 8.5	178 ± 4.5

Parameters reported as mean ± standard deviation.

The igneous rock phosphate used in the study was collected from Phalaborwa located in the Limpopo Province of South Africa (sample reference Palfos 88). The rock phosphate was supplied as a fine ground powder which could all pass through a 0.25 mm sieve. The chemical properties of the rock phosphate were provided through a certificate of analysis produced by an accredited laboratory and availed by Forskor, Paraborwa, South Africa. The rock phosphate sample used in this study had the following chemical properties: P_2_O_5_ – 40.3%; CaO – 54.6%; MgO – 0.26%, cadmium – 1.2 mg kg^−1^, chromium – 18.05 mg kg^−1^, copper – 5.85 mg kg^−1^, lead – 6.05 mg kg^−1^ and zinc – 13.22 mg kg^−1^. The mature epigeic earthworms (*Eisenia fetida*) were obtained from a local supplier in East London, South Africa.

### 2.2 Experimental set up and treatments

The vermicomposting was done in plastic rectangular vermi-reactors measuring 0.5 m × 0.4 m × 0.3 m (*l × w × h*; volume 0.06 m^3^) with perforations on the bottom and the lids. The cow dung and shredded waste paper were mixed i.e. 5.7 kg cow dung and 8.3 kg waste paper, to achieve a C/N ratio of 30 ± 1 following recommendations of Mupondi et al. (2010). Prior to enrichment with rock phosphate, the mixtures were moistened to a moisture content of 60% ± 10, and pre-composted for 1 week to eliminate volatile toxic gases, which are harmful to earthworms (Mupambwa and Mnkeni, 2015). The treatments in this study consisted of different levels of P enrichment applied as igneous rock phosphate i.e. 0%; 2%; 4% and 8% P (dry w/w basis). These treatments were laid in a completely randomized design with each treatment replicated three times. Mature earthworms were introduced into each vermi-reactor at a stocking density of 13.6 g-worm kg^−1^ of biomass (0.75 kg – feed/kg-worm/day) based on recommendations of Ndegwa and Thompson (2000). A total of 14 kg (dry weight) of cow dung-waste paper-rock phosphate mixture was used for all treatments, with no feed being added during the entire vermicomposting period. A subsample was collected from each treatment at 0, 2, 4 and 8 weeks and immediately stored at 4 °C before analysis for microbial biomass carbon, while the other bio-chemical parameters were only analyzed for samples from the 0 and 8 weeks samplings.

### 2.3 Organic chemical analysis

Total C and N was determined using the dry combustion method using a LECO TruSpec C/N auto analyzer (LECO Corporation, USA).

The humic and fulvic acid fractions in the composts were extracted using a method described by Sanchez-Monedero et al. (1996). Extraction was done using 0.1 M NaOH at a ratio of 1: 20 w/v, shaken on a horizontal reciprocating shaker for 4 h and centrifuged at 8000 rpm equivalent to 8.2 × 10^3^ × *g* relative centrifugal force. Half of the extracts were stored for subsequent analysis of total extractable carbon fraction (C_tEX_) and the remainder acidified to pH 2 using concentrated sulphuric acid. The pH adjusted extracts were allowed to coagulate over 24 h at 4 °C before centrifugation at 8.2 × 10^3^ × *g* relative centrifugal force to separate the humic acid (HA) from the fulvic acid (FA) fraction. The fulvic acid (C_FA_) portion in solution and the (C_tEX_) fraction were then analysed for extractable carbon using the dichromate oxidation method. The extractable carbon in the extracts was calculated using Eq. (1), as described by Anderson and Ingram (1993).

(1) $$\text{Organic}\;\text{carbon}\;\left(\text{\%}\right)=\frac{\left(\text{A}\times \text{M}\times 0.003\right)}{\text{g}}\times \frac{\text{E}}{\text{S}\;\;}\times 100$$

**Where:**

**A** = (mL heated blank (HB) − mL sample) × [(mL unheated blank (UB) − mL HB)/mL UB] + (mL HB − mL sample).
**M** = molarity of ferrous ammonium sulphate; **g** = dry soil mass (g); **E** = extraction volume (mL); **S** = digest sample volume (mL).

The C concentration of the humic acid (C_HA_) fraction was then calculated as the difference between the total extractable carbon and the fulvic acid carbon. Humification ratio (HR; Eq. (2)), humification index (HI, Eq. (3)), polymerization index (PI, Eq. (4)) and percent of humic acids (PHa, Eq. (5)), which are indices used for the evaluation of humification level in the compost, were then calculated as shown below, where C is total carbon determined by dry combustion (LECO Corporation, 2003).

(2) $$\text{HR}=\frac{{\text{C}}_{\text{tEX}}}{C}\times 100$$

(3) $$\text{HI}=\frac{{\text{C}}_{\text{HA}}\;}{C}\times 100$$

(4) $$\text{PI}=\frac{{\text{C}}_{\text{HA}}}{{\text{C}}_{\text{FA}}}$$

(5) $$\text{PHa}=\;\frac{{\text{C}}_{\text{HA}}\;}{{\text{C}}_{\text{tEX}}}\times 100$$

The chloroform fumigation–extraction procedure was used for determination of microbial biomass C in compost samples based on the methods of Vance et al. (1987) and as outlined in detail by Mupambwa and Mnkeni (2015).

### 2.4 Inorganic chemical analysis

Mineral nitrogen (NH_4_^+^-N; NO_3_-N and NO_2_-N) was extracted using 0.5 M K_2_SO_4_ (1:10; w v^−1^) (Maynard et al., 2006). The nitrate and ammonium concentrations were determined using a spectrophotometer after development of a yellow colour using 5% salicylic acid in concentrated sulphuric acid, for nitrate and nitrite-N, and a blue colour using salicylate-nitroprusside colorimetric method, for ammonium-N (Okalebo et al. 2002).

Vermicompost samples collected at the start (day 0) and end (day 56) of vermicomposting were subjected to sequential P fractionation using a procedure outlined by Gichangi et al. (2009). Moist compost samples (0.5 g oven-dried basis) were placed in centrifuge tubes with two resin strips (10 mm × 60 mm; Anion 103QDP-EO6295A and Cation CR61CIP-MO6095A supplied by Ionic's inc. Massachusetts) and 30 mL deionized water and shaken at 175 oscillations per minute for 16 h at room temperature. The resin strips were carefully removed and thoroughly rinsed in water. The adsorbed P was then recovered in 20 mL of 0.5 M HCl after shaking for 30 min, and the inorganic P in the HCl extracts was then determined by ascorbic acid method (Kuo, 1996). The compost suspension remaining following removal of the resin strips was centrifuged for 10 min at 1.28 × 10^4^ × *g* relative centrifugal force and the supernatant discarded. The compost residues left in the centrifuge tubes were then sequentially extracted with 30 mL each of 0.5 M NaHCO_3_ (pH 8.5); 0.1 M NaOH and 1.0 M HCl. After sequentially shaking with the extractants for 16 h at 175 oscillations per minute, at each stage, the suspensions were centrifuged at 1.28 × 10^4^ *g for 10 mins. The three supernatants obtained were then used for determination of the NaHCO_3_; NaOH and HCl extractable P using the ascorbic acid method (Kuo, 1996).

For heavy metal i.e. Cr, Cd, Pb, Zn and Cu analysis in both the vermicomposts and earthworm tissues, the samples were digested using acids as outlined by Faithfull (2002). Following drying and grinding (<1 mm) of the composts, the samples were digested using an *aqua regia* solution. The earthworms were placed on wet filter paper for a period of 24 h to allow the depuration of their gut contents and enable accurate estimation of the heavy metals in the tissues of the earthworms. The earthworms were then washed in distilled water, dried on paper towels, killed by freezing, dried, ground and then digested using an *aqua regia* solution. The concentration of heavy metals in the digested vermicompost and earthworm samples was then determined using an atomic absorption spectrophotometer (iCE 3300 Atomic Absorption Spectrometer, Thermo Scientific, USA).

### 2.5 Statistical analysis

A one way analysis of variance was performed on the data to identify differences between treatments on all parameters measured, for each sampling, though for MBC, a two way ANOVA was performed. The JMP version 12.0.1 statistical software (SAS Institute, Inc., Cary, North Carolina, USA, 2010) was used for all statistical analysis. Where significant differences were observed, Fishers Protected Least Significant Difference was used to separate the means at *p* < 0.05. All graphs were plotted using MS Excel 2013; which was also used for determination of the regression equations.

## 3 Results and discussion

### 3.1 Effects of rock phosphate incorporation level on compost maturity and MBC

**Fig. 1:**
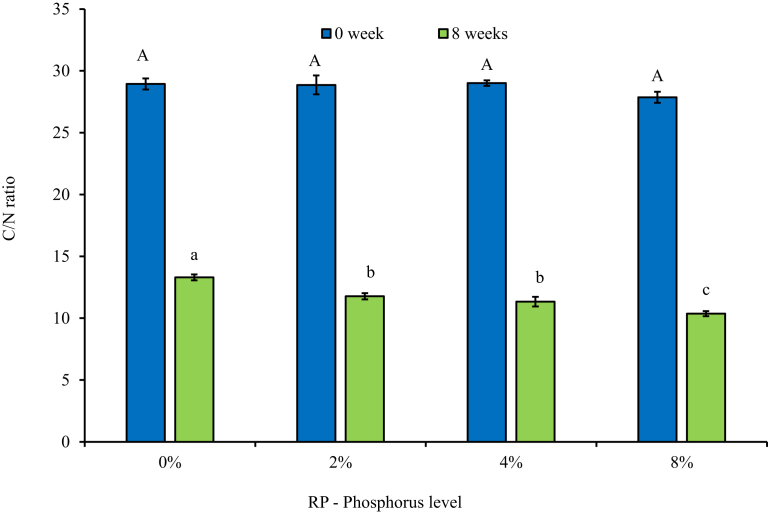
Changes in C/N ratio following 8 weeks of vermicomposting cow dung-paper mixture amended with increasing levels of phosphorus from igneous rock phosphate. *Error bars represent standard deviation. *Different uppercase letters indicate significant differences (*P* < 0.05) between treatments at week 0; whilst different lowercase letters indicate significant differences (*P* < 0.05) between treatments at week 8. *Data for the figure is provided as Supplementary data A.

Compost maturity was determined based on changes in C/N ratio and humification parameters, which are among the key parameters for qualifying vermicompost maturity (Bernal et al., 2009; Raj and Antil, 2011). Across all maturity parameters measured in this study, there was a progressive increase in compost maturity as the levels of rock phosphate inclusion increased. According to Bustamante et al. (2008) and Bernal et al. (2009), a mature compost should have a C/N ratio of less than 15, with a C/N ratio of less than 10 being preferable. Though all treatments managed to reach maturity based on the above criteria, incorporation of rock phosphate at 2% and 8% P resulted in a 12% and 22% significantly (*P* < 0.001) lower final C/N ratio, respectively, compared to the control (Fig. 1). The carbon to phosphorus ratio has been identified as having an impact on biodegradation as microbial activity and growth is optimized at narrower C/P ratios (Mupambwa and Mnkeni, 2018). Brown et al. (1998) reported that a C/P ratio of between 120:1 and 240:1 is necessary for optimal biodegradation. This could explain why biodegradation, measured as the decrease in C/N ratio, increased as the C/P ratio narrowed with increased levels of RP inclusion from the high level of 237:1 in the control with no P added.

**Fig. 2:**
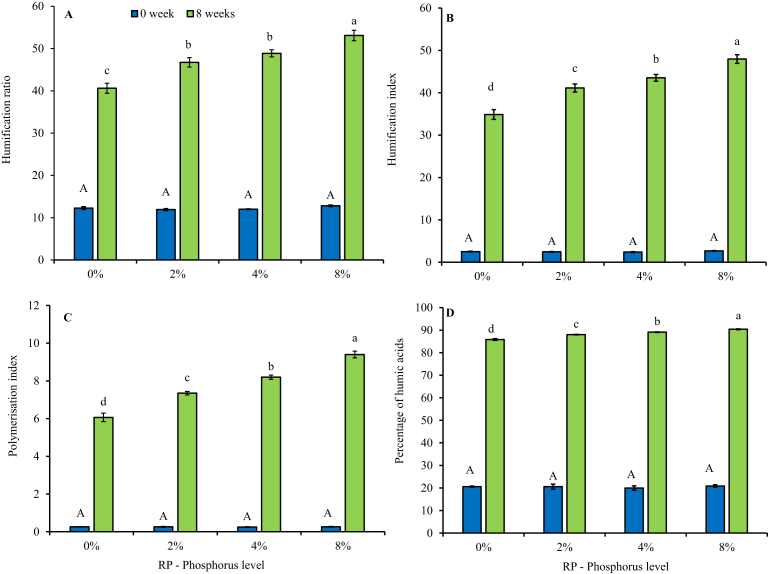
Changes in (a) humification ratio, (b) humification index, (c) polymerization index and (d) percentage of humic acids in cow dung-paper mixture amended with increasing levels of phosphorus from igneous rock phosphate. *Error bars represent standard deviation. *Different uppercase letters indicate significant differences (*P* < 0.05) between treatments at week 0; whilst different lowercase letters indicate significant differences (*P* < 0.05) between treatments at week 8. *Data for the figure is provided as Supplementary data B.

A similar trend was observed on the humification parameters, where the amendment of rock phosphate into the cow dung – waste paper mixtures resulted in a complementary linear increase of these parameters (Fig. 2). According to Bernal et al. (2009), a mature compost should have a HR > 3.5; PHa > 50 and PI > 1.0, and all treatments with or without addition of rock phosphate resulted in humification parameters that satisfied these criteria.

What was interesting to note in this study was that, though all treatments including the control without RP managed to reach maturity after 8 weeks based on C/N ratio and humification parameters, the higher the RP inclusion, the higher the maturity parameters. Amendment of organic materials with rock phosphate did not change the initial C/N ratio of the materials significantly (Fig. 1), though it changes the volume of organic materials within each treatment depending on the weight of the rock phosphate. These differences in organic materials available in each treatment could explain the slight differences in biodegradation of organic matter indicated by the differences in C/N ratio, as the level of RP increases. Since the control in this study also had earthworms, results from this study indicate that inclusion of igneous RP may not significantly influence changes in C/N ratio as observed by Unuofin et al. (2016), though RP inclusion significantly influenced humification of the vermicomposts. It may be of interest to determine if this C/P ratio could be influenced by the solubility of the P source.

**Fig. 3:**
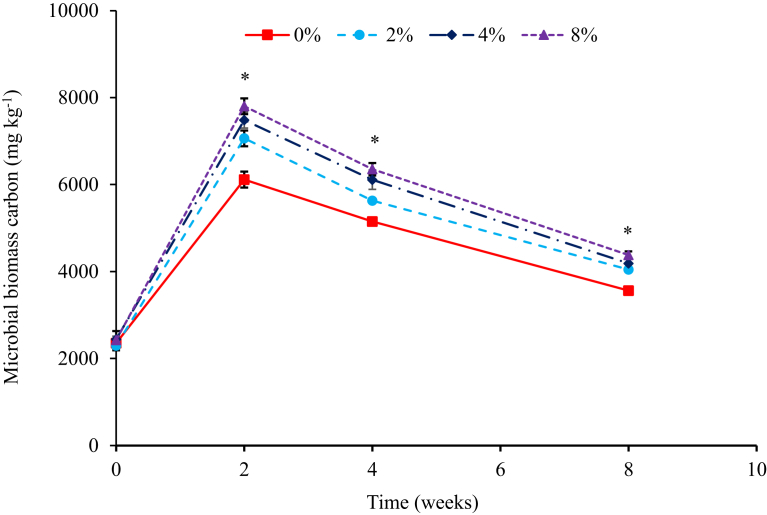
Changes in MBC during vermicomposting of cow dung-paper mixtures amended with increasing levels of phosphorus from igneous rock phosphate. Error bars represent standard deviation. *Indicates significant differences at (*P* < 0.05). *Data for the figure is provided as Supplementary data C.

Across all treatments, microbial biomass carbon peaked at 2 weeks and thereafter, decreased consistently until the end of vermicomposting at 8 weeks (Fig. 3). There was a significant difference (*P* < 0.001) between all treatments, across all 8 weeks of vermicomposting, with the control treatment having the lowest microbial biomass carbon (MBC). The consistent differences between treatments across all weeks could explain the significant interaction (*P* < 0.001) between rock phosphate incorporation level and time. The decrease in MBC across all treatments is due to rapid decomposition of organic matter, which deplete resources for microbial growth, in the presence of earthworms (Pramanik et al., 2011; Pramanik and Chung, 2011; Mupambwa and Mnkeni, 2015). It was also interesting to note that, the higher the level of rock phosphate amendment, the higher the MBC across all weeks, which correlates with observations in the compost maturity data (Fig. 3). The higher MBC at higher P rock rates suggested that increasing P resulted in greater biodegradation, mainly due to optimized C/P ratio as described by Mupambwa and Mnkeni (2018), resulting in greater humification and maturity of the composts. The high MBC values were also consistent with those reported by several researchers evaluating the vermi-degradation of different substrates such as fly ash Mupambwa and Mnkeni (2015); pig manure Aira et al. (2007); industrial sludge and poultry manure (Pramanik et al., 2011); among others.

### 3.2 Effects of rock phosphate incorporation on nutrient dynamics

#### 3.2.1 Phosphorus fractions

**Fig. 4:**
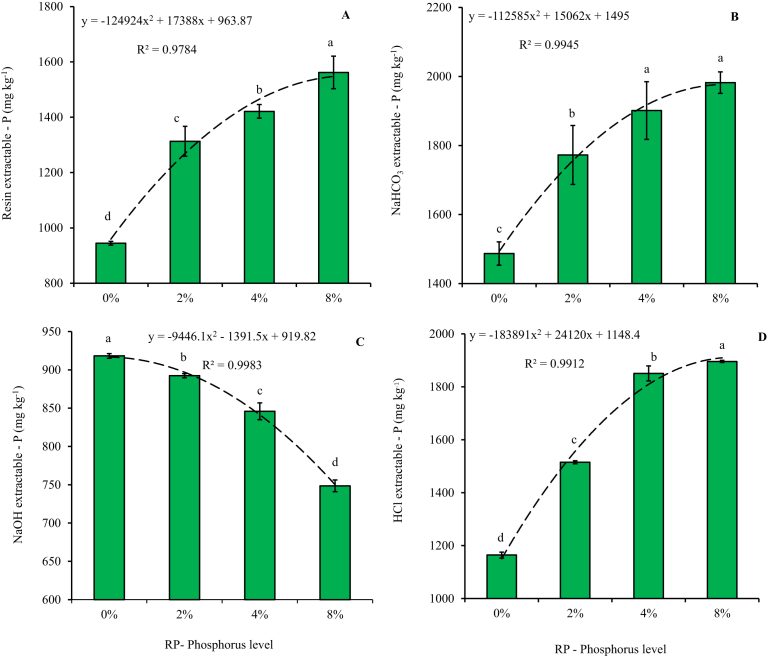
Phosphorus fractions (a) resin extractable P, (b) sodium carbonate extractable P, (c) sodium hydroxide extractable P, (d) hydrochloric acid extractable P; after 8 weeks of vermicomposting of cow dung-paper mixtures amended with increasing levels of phosphorus from igneous rock phosphate. Dotted continuous line shows a second order regression equation showing the changes in phosphorus concentration under increasing RP amendment levels. Error bars represent standard deviation. *Data for the figure is provided as Supplementary data D.

Four phosphorus fractions were determined, which simulated different P availability indices in the compost and soil. Phosphorus in soil is found in different pools i.e. organic and inorganic, and sequential P fractionation, as done in our study, can be used to separate the different soil P forms which is critical in assessing the fluxes between P forms (Alamgir et al., 2012). There were significant differences (P < 0.001) observed between treatments on all the P fractions determined. Though in the beginning the level of resin, NaHCO_3_; NaOH and HCl extractable P were not significantly different, there were significant differences observed consistent with the RP amendment level after 8 weeks of vermicomposting (Fig. 4). Across all the four fractions, as the level of RP amendment increased from 0 to 8%, the final concentration of resin, NaHCO_3_ and HCl significantly increased (Fig. 4). According to Odongo et al. (2007), resin P represents a pool from which plants readily take P up. Amending the cow dung – waste paper mixture with 2%, 4% and 8% P as rock phosphate, resulted in a 39%; 50% and 65% more resin extractable P, respectively, compared to the control with no rock phosphate. Similarly, the bicarbonate extractable P, which represent the bioavailable or labile P fraction (Adhami et al., 2014), increased consistently by 19%; 28% and 33% following 2%, 4% and 8% RP application, respectively, compared to the control. The HCl extractable P fraction in the vermicompost showed the greatest increase across all treatments after 8 weeks compared to the starting values at 0 weeks (Fig. 4). The only fraction that showed a decrease, as the level of RP increased was the NaOH extractable P fraction, which represents the inorganic Al-P and Fe-P fractions within the vermicompost (Adhami et al., 2014). The results of this study are consistent with those of Adhami et al. (2014), in which addition of RP enhanced the available fraction of phosphorus following vermicomposting, compared to the control with no RP. What was also interesting to observe was the continued increase in P fractions as the level of rock phosphate increased. This means that optimization of RP incorporation, based on P release and not vermicompost maturity may be necessary. In a similar study, Unuofin et al. (2016) reported continued increase in P extractability as the RP level increased up to only 4%. However, our study seems to show that beyond the 4% reported by Unuofin et al. (2016), an optimum level in terms of P release tends to be observed around 7% RP incorporation during vermicomposting as indicated by the second order regression equation.

#### 3.2.2 Nitrates, nitrites and ammonium

**Fig. 5:**
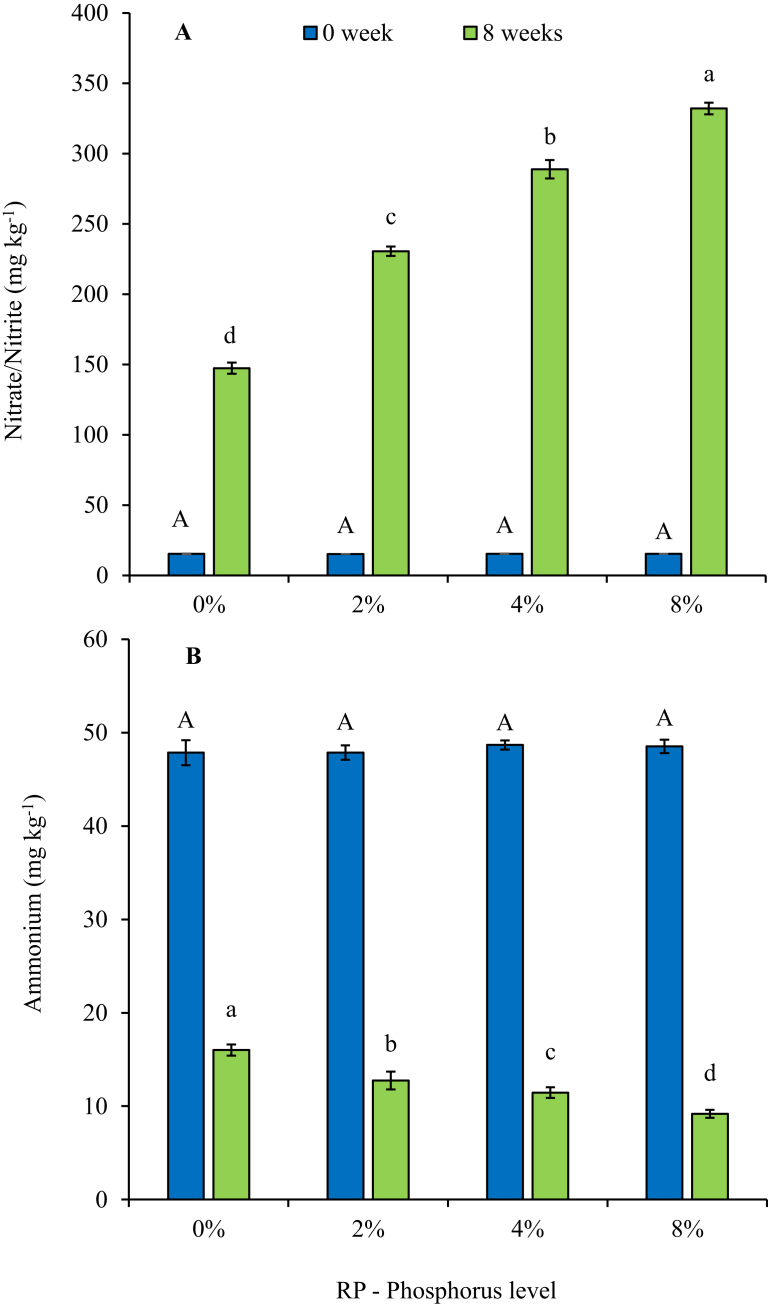
Changes in (a) nitrate/nitrite – N and (b) ammonium-N during vermicomposting of cow dung-paper mixture with increasing phosphorus levels from igneous rock phosphate. *Error bars represent standard deviation. *Different uppercase letters indicate significant differences (*P* < 0.05) between treatments at week 0; whilst different lowercase letters indicate significant differences (*P* < 0.05) between treatments at week 8. *Data for the figure is provided as Supplementary data E.

Though rock phosphate contains very insignificant levels of nitrogen, its influence on N mineralization when incorporated into cow dung – waste paper mixture during vermicomposting was determined in this study. There was an almost linear increase in final nitrate/nitrite levels, as the incorporation rate of rock phosphate increased from 0% to 8%, whilst the ammonium levels showed a corresponding but decreasing trend as the RP levels increased (Fig. 5). It is noteworthy that following 8 weeks of vermicomposting, incorporation of rock phosphate at 2%; 4% and 8% significantly increased (P < 0.001) nitrates levels by 56%; 96% and 125%, respectively, relative to the control. Though all treatments significantly reduced the levels of ammonium following 8 weeks of vermicomposting, the highest reduction was observed where there was 8% RP and the least where there was no RP added. The enhanced N mineralization could be attributed to an increased biodegradation as indicated by the higher MBC, supported by increased availability of essential P from the RP in the vermicomposts. Bhattacharya and Chattopadhyay (2004) made similar observations when inorganic fly ash, rich in P, was incorporated during vermicomposting, reporting high levels for nitrogen fixing microbes compared to treatments with the organic material only.

### 3.3 Influence of rock phosphate incorporation level on compost and earthworm heavy metals concentrations

Rock phosphate being terrestrial in origin, also contains some toxic heavy metals, which can be solubilized or immobilized during vermicomposting. However, only few studies have examined the contribution of igneous rock phosphate on heavy metal enrichment of vermicomposts (Goswami et al., 2016; Mupondi, 2010). Elements like Cd in RP are usually bound to the P in apatite structure, thus they can be simultaneously released with P solubilization (Mupondi, 2010). In this study, the concentration of total heavy metals (Cr, Cd, Cu, Pb and Zn) were monitored at the beginning and end of vermicomposting of cow dung and waste paper mixtures enriched with RP (Table 2). On average, relative to the control, incorporation of rock phosphate from 2 to 8% resulted in a respective 8.5; 2.3; 2.9; 5.4 and 3.2 times more Cr, Cu, Cd, Pb and Zn, relative to what was measured at 0 weeks before vermicomposting. Though RP addition at 0 weeks resulted in significantly higher contents of heavy metals, all of the concentrations were below the maximum permissible concentration of potential toxic elements in soils, as recommended by the United Kingdom Regulations (1989) cited by Muchuweti et al. (2006). Following 8 weeks of vermicomposting, the concentration of all heavy metals significantly decreased by close to 50% in all treatments including the control (Table 2). Reductions of 40%; 35%; 35%; 40% and 45% in Cr, Cu, Cd, Pb and Zn, respectively, were observed in the 8% RP treatment after 8 weeks of vermicomposting. These results are in agreement with those of Goswami et al. (2016), who observed significantly higher removal efficiency of up to 40% in a vermicompost with *E. fetida* compared to the compost without any earthworms. The results support the idea that during vermicomposting, earthworms are responsible for the active bio-accumulation of toxic heavy metals through a metal inducible protein called metallothionein, which forms organo-metallic complexes with the heavy metals, thus reducing their availability within the vermicomposts (Bhattacharya and Kim, 2016).

**Table 2:** Changes in heavy metals during vermicomposting of cow dung-paper composts with increasing phosphorus from rock phosphate.

RP – Phosphorus level (RPPL)	Cr		Cu		Cd		Pb		Zn	
	mg kg^−1^									
	0 week	8 weeks	0 week	8 weeks	0 week	8 weeks	0 week	8 weeks	0 week	8 weeks
0%	4.56d^a^	2.74d	5.79d	3.76d	1.09d	0.71d	2.43d	1.46d	8.49d	4.67d
2%	20.66c	12.40c	10.85c	7.05c	2.35c	1.53c	8.33c	5.00c	20.28c	11.16c
4%	37.56b	22.53b	12.90b	8.38b	3.13b	2.03b	13.51b	8.11b	28.53b	15.69b
8%	57.97a	34.78a	15.66a	10.18a	4.05a	2.64a	17.82a	10.69a	32.74a	18.00a

a Different lower case letters within each column indicate a significant difference between the mean values at *P* < 0.05. Data for the table is provided as Supplementary data F.

The above observations are supported by heavy metal concentrations in earthworm tissues that show that as the level of RP amendment increased from 0% to 8%, the concentrations of all measured heavy metals significantly increased within the earthworm tissues (Table 3). Inclusion of RP to supply 8% phosphorus resulted in 2.81 times more Cr; 2.52 times more Cu; 2.76 times more Cd; 6.3 times more Pb and 3.58 times more Zn; within the earthworm tissues; relative to the control at the end of the experiment. According to Usmani et al. (2017), the metal accumulation ability in earthworms can be evaluated based on the concentration of the metals within the earthworm tissues divided by the metal concentration in the vermicomposts. Bioaccumulation factors (BAF) of greater than 1 indicate higher concentrations of heavy metals within the earthworm tissues relative to the compost, which was not the case in our study. It would be interesting to evaluate the metal bioaccumulation efficiency of different earthworm species during rock phosphate vermicomposting.

**Table 3:** Changes in concentrations of heavy metals in earthworm tissues at start and end of vermicomposting.

RP – Phosphorus level (RPPL)	Cr		Cu		Cd		Pb		Zn	
	mg kg^−1^									
	0 week	8 weeks	0 week	8 weeks	0 week	8 weeks	0 week	8 weeks	0 week	8 weeks
0%	0.53a^a^	1.64d	0.85a	1.32d	0.30a	0.30d	0.39a	0.56d	1.44a	2.18d
2%	0.57a	2.68c	0.85a	2.47c	0.29a	0.53c	0.40a	1.75c	1.43a	5.21c
4%	0.58a	3.78b	0.83a	2.85b	0.30a	0.64b	0.42a	2.73b	1.44a	6.90b
8%	0.57a	4.62a	0.87a	3.33a	0.29a	0.83a	0.37a	3.53a	1.43a	7.80a

a Different lower case letters within each column indicate a significant difference between the mean values at *P* < 0.05. Data for the table is provided as Supplementary data G.

## 4 Conclusions

The results of this study showed that during vermicomposting with *E. fetida*, addition of rock phosphate could significantly enhance microbial biomass carbon, an indicator of enhanced biodegradation, compost stabilization and nitrogen mineralization compared to the control without rock phosphate. The influence of RP amendment on biodegradation indicated the importance of the need to optimize the C/P ratio and not only C/N ratio during vermicomposting. Incorporation of rock phosphate -P at 2–8%, significantly increased final P bioavailability, with an optimum P release being observed around 7% RP. Vermicomposting with *E. fetida* significantly reduced heavy metals by up to 45%, to levels below the maximum permissible concentration of potential toxic elements in soils after 8 weeks of vermicomposting. However, though reduced heavy metal concentrations were observed under earthworms, this study did not determine earthworm survival under the various RP levels, thus this reduction maybe due to other unknown biological processes and not entirely due to earthworm activity. Furthermore, our study only focused on *Eisenia fetida* earthworm species, thus it is not known whether similar observations will be made when a different species is used for vermicomposting of organic materials amended with igneous rock phosphate.

## Declarations

### Author contribution statement

Lushian Tapiwa Mupondi: Conceived and designed the experiments; Performed the experiments; Analyzed and interpreted the data; Contributed reagents, materials, analysis tools or data; Wrote the paper.

Pearson Nyari Stephano Mnkeni: Conceived and designed the experiments; Analyzed and interpreted the data; Contributed reagents, materials, analysis tools or data; Wrote the paper.

Pardon Muchaonyerwa: Conceived and designed the experiments; Wrote the paper.

Hupenyu Allan Mupambwa: Analyzed and interpreted the data; Wrote the paper.

### Funding statement

This work was supported by a doctoral bursary to the late Dr. LT. Mupondi by the National Research Foundation of South Africa.

### Competing interest statement

The authors declare no conflict of interest.

### Additional information

No additional information is available for this paper.
